# Role of Magnetic Resonance Perfusion Imaging in Acute Stroke: Arterial Spin Labeling Versus Dynamic Susceptibility Contrast-Enhanced Perfusion

**DOI:** 10.7759/cureus.23625

**Published:** 2022-03-29

**Authors:** Ganesan Gopinath, Malik Aslam, Palaniswamy Anusha

**Affiliations:** 1 Radiology, Panimalar Medical College Hospital and Research Institute, Chennai, IND; 2 Radiology, Saveetha Medical College, Chennai, IND

**Keywords:** perfusion diffusion mismatch, magnetic resonance imaging (mri), dynamic susceptibility contrast (dsc), arterial spin labelling (asl), acute stroke, perfusion imaging

## Abstract

Introduction: The role of perfusion neuroimaging in managing cases of acute ischemic stroke (AIS) is to identify ischemic penumbra and regions of hypo-perfusion, which can be salvaged. Dynamic susceptibility contrast (DSC) perfusion imaging techniques have been the main magnetic resonance imaging (MRI) perfusion techniques used to identify AIS. Arterial spin labelling (ASL) is an alternative non-invasive perfusion technique, which permits tissue perfusion measurement without any need for administration of exogenous contrast agents. The objective was to compare the diagnostic accuracy of ASL perfusion MRI versus DSC enhanced perfusion MRI in detecting perfusion-diffusion mismatch of varying volumes in acute ischemic stroke.

Materials and methods: A hospital-based observational cross-sectional study was done in a tertiary care institute in Tamil Nadu between December 2018 to October 2019. Fifty-five subjects aged more than 18 years referred to the Radio-diagnosis department (less than 24 hours since the onset of weakness) for emergency assessment of suspected acute stroke were subjected to MRI stroke scan protocol. Then AIS cases were evaluated with ASL and DSC perfusion-weighted imaging. The collected data was entered in Excel (Microsoft, Redmond, WA, USA). IBM SPSS version 22 (IBM Corp., Armonk, NY, USA) was used for statistical analysis. Receiver operating characteristic (ROC) analysis was done to assess the predictive validity of ASL in predicting DSC mismatch. The diagnostic accuracy of ASL was the primary outcome variable. P-value < 0.05 was considered statistically significant.

Results: Forty-four subjects confirmed as stroke were included in the final analysis. Their mean (±SD) age was 53.84 (±10.80) years. 72.7% were males. The majority (53.8%) presented during the acute stage of cerebral infarction (53.8%). The majority (45.5%) had hemiplegia followed by aphasia (27.3%). The major vascular territory involved was the middle cerebral artery (54.5%). The sensitivity, specificity, positive predictive value, and negative predictive value of ASL (non-contrast) in predicting DSC (contrast) mismatch was found to be 71.43%, 78.57%, 83.33%, and 64.71% respectively.

Conclusion: ASL MR has the potential to replace MRI DSC perfusion in the future imaging diagnostic work-up for stroke. However, further studies are required to validate its role as the first-line imaging for stroke therapy.

## Introduction

Globally, stroke is one of the leading causes of death and disability. It is the second most common cause of death worldwide and also the third most common cause of disability and death combined [1,2]. It is responsible for 11.6% of total deaths and 5.7% of total disability-adjusted life years (DALYs) worldwide according to the systematic analysis of the Global Burden of Diseases, Injuries and Risk factors (GBD) 2019 Study for the period 1990 to 2019 [1]. Stroke is a major cause of premature death and disability in low- and middle-income countries like India [3]. There has been a substantial increase in deaths and incidence of cases from 1990 to 2019 with the highest loss of DALYs in low-income group countries [1]. According to a meta-analysis of community-based surveys in India, the prevalence rate of stroke varied between 44.54 to 150 per 100,000. The 30-day case fatality rate ranged between 41% to 42% in urban India and between 18% to 46% in rural India [4].

Stroke is a medical emergency characterised by abrupt neurological outburst due to impaired perfusion via the blood vessels of the brain [5]. The sooner the patient receives treatment, the less damage is likely. Perfusion neuro-imaging plays a major role in acute ischemic stroke (AIS) cases to evaluate the reduction of regional blood flow, identify ischemic penumbra and regions of hypo-perfusion, which can be rescued with thrombolytic therapy or with endovascular recanalization therapy [6-8]. Perfusion MRI techniques have the ability to differentiate between irreversibly infarcted brain tissues and potentially salvageable brain tissues resulting in a better selection of patients for therapy [9,10]. Recent advances in pulse sequence design and widespread availability of higher field (1.5 Tesla/3 Tesla) MRI scanners have resulted in increased adoption of perfusion neuro-imaging techniques by improving the signal-to-noise ratio [11,12]. 

Dynamic susceptibility contrast (DSC) perfusion imaging techniques have been the main MRI perfusion techniques used to identify AIS. A time series of fast T2 weighted images is obtained after administration of gadolinium contrast agent [13,14]. It provides “the time to the maximum of the tissue residual function” (Tmax) to define hypoperfusion. DSC has been used to identify subjects who can benefit from reperfusion beyond the conventional window time and subjects whose timeline of onset of symptoms is not known. But their limitation is nephrogenic systemic fibrosis risk with use of contrast agents [15]. Their use is limited in patients with contraindications to intravenous contrast material administration such as renal failure. Hence there is a need for alternative techniques to detect perfusion deficits without the need for administration of contrast.

Arterial spin labelling (ASL) is an alternative non-invasive perfusion technique, which permits tissue perfusion measurement without any need for administration of exogenous contrast agents. It works by tagging the water magnetically in inflowing blood [9,14,16-18]. Cerebral blood volume (CBV) and cerebral blood flow (CBF) are the major parameters used in perfusion imaging. The primary metric that has been in use in brain perfusion imaging such as in brain tumors is CBV from DSC MR perfusion while CBF, predominantly from ASL, has been a developing focus [19]. The perfusion contrast is acquired due to difference in magnetization, brought about by labelled spins exchange at the level of brain tissue in comparison with a non-labelled control image.

Evidence from a cohort study has concluded responses to thrombolytic therapy can be predicted by specific mismatch patterns across perfusion and diffusion lesions [20]. Perfusion weighted imaging (PWI) with MRI or CT in AIS can rule in acute ischemia besides ruling out stroke mimics. Although diffusion-weighted imaging can confirm AIS diagnosis, it can miss ischemia of moderate degree and in early stages. Previous studies done in other countries have shown the potential of ASL to detect perfusion deficits in acute stroke [6,21-24]. ASL has not been evaluated in Indian settings by comparing with DSC at the time of initiation of the study and remains unclear. Hence the present study was carried out with the objective of comparing the diagnostic accuracy of ASL perfusion MRI versus DSC-enhanced perfusion MRI in detecting perfusion-diffusion mismatch of varying volumes in acute ischemic stroke.

## Materials and methods

An observational cross-sectional study was done in the Radio-Diagnosis & Imaging Sciences Department, Saveetha Medical College & Hospital, Thandalam, Chennai District, Tamil Nadu, India. The study was conducted after getting clearance from Saveetha University's institutional ethics committee (Ethical clearance certificate no. SMC/IEC/2018/11/228). The study period was from December 2018 to October 2019. Informed consent was obtained prior to the study after explaining the objectives, procedure and investigations in detail to the patient/attenders.

The study population included patients with clinical suspicion of acute ischemic stroke, referred to the department of Radio-Diagnosis for emergency assessment of suspected acute stroke during the study period. Based on the reference study on ASL vs DSC perfusion imaging for stroke diagnosis, the sensitivity of ASL technique was calculated as 61%. Taking it as the prevalence, with limit of accuracy as 14% and with the Z value of 1.96, the sample size calculated was 47. About 16% of the sample size was added for refusal to participate in the study. Hence, the minimum sample size arrived at was 55. Purposive sampling was done till the desired sample size was reached. The sampling was purposive, as only clinically suspected/diagnosed cases of CVA with plain CT brain ruling out hemorrhagic stroke were considered for the study.

The inclusion criteria were patients who were clinically suspected of hyperacute/acute ischemic stroke (less than 24 hours since onset of weakness) and aged more than 18 years. All patients having 1) cardiac pacemakers, prosthetic heart valves, cochlear implants or any metallic implants, 2) patients with hemorrhagic stroke, patients having history of claustrophobia, 3) patients with a glomerular filtration rate of less than 30 ml/min to avoid contrast related complications and 4) patients with subacute to chronic ischemic stroke were excluded from the study. Patients who did not give consent to participate in the study were also excluded.

Fifty-five patients satisfying the inclusion and exclusion criteria were imaged with a fast stroke protocol MRI scan. MRI Brain stroke protocols (fluid attenuated inversion recovery [FLAIR], DWI, ASL and DSC) were done in the following sequence: DWI and FLAIR followed by ASL PWI, followed by DSC PWI. Diagnosis on MRI was made with background of clinical context. The study was conducted on the Philips Multiva 1.5 Tesla MRI (Philips, Amsterdam, Netherlands) using dedicated 16-channel head coils. Fast stroke scan protocol consists of MRI sequences of FLAIR, DWI, ASL and DSC with a scan time of less than eight minutes. Firstly, ASL PWI scans were done by a pseudo-continuous ASL pulse sequence with background suppressed three-dimensional gradient and spin echo readout (field of view = 24 x 24 x 10 cm, matrix = 80 × 80, Number of slices = 11, GRAPPA factor - SENSE (NO), TR = 3944 ms, TE = 14 ms, number of slabs = 1) and scan time of four minutes. Followed by DSC PWI scans were acquired using a gradient-echo echo planar imaging sequence (TR = 1750 ms, TE = 40 ms, field of view = 22x x22 x 11 cm, matrix = 128 × 128, number of slices = 25, slice thickness - 5 mm, scan time = 80 seconds) with gadolinium contrast agent (0.1 mmol/kg) bolus administered intravenously. The MRI protocol includes DWI, gradient recalled echo, fluid-attenuated inversion recovery, and PWI sequences. Perfusion deficit was defined as an area with visually significant increased time to peak (TTP) on DSC and with decreased perfusion signal on ASL when compared with the surrounding brain tissue and contralateral hemisphere. Images were in a random order and blinded to clinical information and were interpreted for image quality, diffusion-perfusion mismatches, visual comparison between ASL (non-contrast, both colour and grey maps) and DSC (contrast) perfusion images.

Statistical analysis

The collected data was entered in Excel (Microsoft, Redmond, WA, USA). For statistical analysis, IBM SPSS version 22 (IBM Corp., Armonk, NY, USA) was used. For describing categorical variables, frequency and proportion was used. Mean and standard deviation was used to describe continuous variables. Chi square test was used to test statistical significance of cross tabulation between categorical variables. Receiver operating characteristic (ROC) analysis was done to assess predictive validity of ASL (non-contrast) in predicting DSC (contrast) mismatch. The sensitivity, specificity, predictive values and likelihood ratios along with their 95% CI were presented for the ROC analysis. For statistical significance, a P value of < 0.05 was considered.

## Results

A total of 44 out of the 55 subjects were confirmed as stroke based on MRI scan stroke protocol on the background of clinical context and were included in the study. The mean (±SD) age of the study population was 53.84 (±10.80) years with a range of 29 to 74 years among which the majority were between 41 to 60 years. 27.3% were females and 72.7% were males. The mean (±SD) time from onset of clinical symptoms to the time of scan was 6.78 (±2.10) hours. The majority of the patients presented during the acute stage of cerebral infarction (53.8%) and the remaining 19 cases (43.2%) were in the hyperacute stage. The majority of the cases had hemiplegia (45.5%) followed by aphasia (27.3%) and so on as shown in Table 1.

**Table 1:** Baseline characteristics of the study population (N=44)

Baseline characteristics		Summary statistics
Mean (±SD) Age in years		53.84±10.80
Age group	≤30	2 (4.5%)
	31-40	2 (4.5%)
	41-50	14 (31.8%)
	51-60	15 (34.1%)
	61-70	6 (13.6%)
	≥71	5 (11.4%)
Gender	Female	12 (27.3%)
	Male	32 (72.7%)
Mean (±SD) time from onset of clinical symptoms to the time of scan in hours		6.78 ± 2.10
Duration of stroke	Acute	25 (56.8%)
	Hyper Acute	19 (43.2%)
Clinical diagnosis	Aphasia	12 (27.3%)
	Gait Disturbance	2 (4.5%)
	Headache	2 (4.5%)
	Hemiplegia	20 (45.5%)
	Visual Disturbances	8 (18.2%)

Among 44 cases, 9.1% suffered from bilateral infarction, 50% suffered from right-sided infarction and 40.9% suffered from left-sided infarction. The most commonly involved major vascular territory was the middle cerebral artery (54.5%), followed by the anterior cerebral artery (29.5%) and posterior cerebral artery (16.0%) (Table 2).

**Table 2:** Characteristics of infarcts in the study population (N=44) ACA: Anterior cerebral artery MCA: Middle cerebral artery PCA: Posterior cerebral artery

Characteristics of infarcts		Frequency (%)
Side	Bilateral	4 (9.1%)
	Left	22 (50.0%)
	Right	18 (40.9%)
Vascular territory	ACA	13 (29.5%)
	MCA	24 (54.5%)
	PCA	10 (16.0%)

Both ASL and DSC were done in 35 patients. Among 21 patients with DSC perfusion mismatch, ASL perfusion mismatch was detected in 15 (71.5%) patients. Patients with ASL mismatch were 9.17 times more likely to be detected as DSC mismatch as compared to patients without ASL mismatch (p=0.004) (Table 3).

**Table 3:** Comparison of perfusion mismatch between ASL and DSC in acute infarcts ASL: Arterial Spin Labelling DSC: Dynamic Susceptibility Contrast

ASL perfusion mismatch	DSC perfusion mismatch		Odds ratio (95% CI)	P-value
	Yes (n=21)	No (n=14)		
Yes	15 (71.5 %)	3 (21.5%)	9.17 (1.87 -44.92)	0.004
No	6 (28.5 %)	11 (78.5%)		

**Figure 1:**
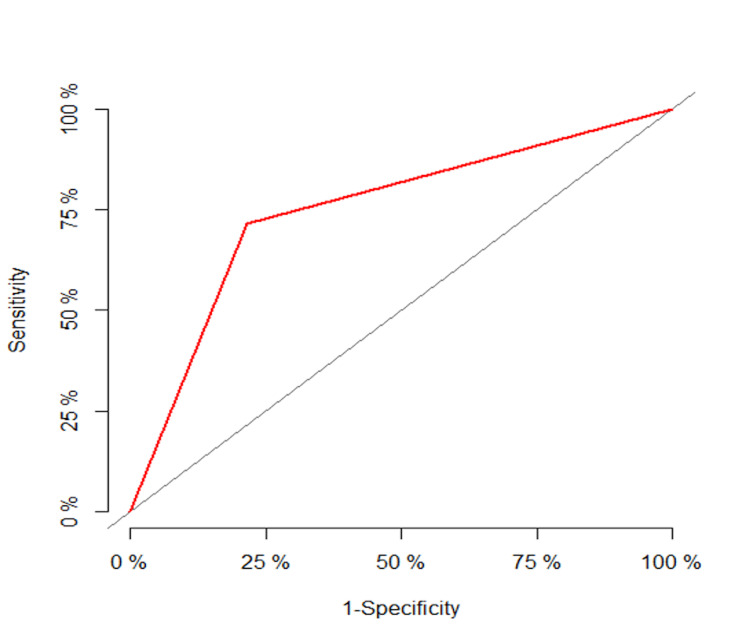
ROC curve for predictive validity of ASL (non-contrast) in predicting DSC (contrast) mismatch ASL: Arterial Spin Labelling DSC: Dynamic Susceptibility Contrast ROC: Receiver Operating Characteristic

The sensitivity, specificity, positive predictive value, and negative predictive value of ASL (non-contrast) in predicting DSC (contrast) mismatch was found to be 71.43%, 78.57%, 83.33%, and 64.71% respectively. The positive likelihood ratio and negative likelihood ratio of ASL (non-contrast) in predicting DSC (contrast) mismatch were 3.33 and 0.36 respectively (Table 4, Figure 1).

**Table 4:** Predictive validity of ASL (non-contrast) in predicting DSC (contrast) mismatch ASL: Arterial Spin Labelling DSC: Dynamic Susceptibility Contrast

Statistics	Value	95% CI
Sensitivity	71.43%	47.82% to 88.72%
Specificity	78.57%	49.20% to 95.34%
Positive Likelihood Ratio	3.33	1.18 to 9.42
Negative Likelihood Ratio	0.36	0.18 to 0.75
Positive Predictive Value	83.33%	63.89% to 93.39%
Negative Predictive Value	64.71%	46.92% to 79.18%

## Discussion

The present study investigated diagnostic accuracy of ASL perfusion MRI versus DSC enhanced perfusion MRI in detecting perfusion-diffusion mismatch in 55 patients clinically suspected of stroke. Forty-four (80%) patients suffered from infarction. Among the 44 patients with infarcts, 35 cases (80%) suffered from acute infarct and the remaining 10 cases (20%) suffered from ischemic stroke mimics. Both ASL and DSC were done in 35 patients. Twenty-one patients had DSC perfusion mismatch while ASL perfusion mismatch was detected in 15 subjects. The sensitivity and specificity of ASL in predicting DSC mismatch was fairly good at 71.43% and 78.57% respectively. Previous studies done in other countries have shown the potential of ASL to detect perfusion deficits in acute stroke [6,21-24].

ASL is a non-ionising and a noninvasive MR perfusion technique that can visualise perfusion deficits without the use of contrast agents. Hence it is suitable for subjects with renal insufficiency and subjects requiring repeated follow-ups. It was first used in 1992 by Williams et al. in rat brain. Since then, it has been primarily used as a research technique [25].

Its use had been historically restricted due to the low intrinsic signal-to-noise ratio. An improved pseudocontinuous labeling scheme has improved the signal-to-noise ratio [14,25,26]. CT perfusion or contrast magnetic resonance perfusion has been considered the gold standard in showing ischemic penumbra. Non-contrast magnetic resonance techniques have inherent disadvantages and are considered less useful. Current sequences on higher magnetic field strength systems can constantly produce higher quality perfusion images using ASL.

Both ASL and DSC were done in 35 patients with DSC perfusion mismatch detected in 21 patients; ASL detected 15 of these perfusion deficits and ASL missed six lesions. In our study we found out that ASL perfusion was able to diagnose perfusion mismatches in predominantly middle cerebral artery (MCA) territorial ischemic infarcts.

In our study, the most affected vascular territory was the MCA, which accounted for 54.5% (24 cases) of all infarcts. It was further observed that the left MCA (58.5%, 14 patients) was more commonly involved. In subjects with perfusion deficit in DSC but normal ASL, the reasons could be small perfusion deficit area/territory of involvement, suboptimal image quality in view of poor tagging of RBC likely secondary to bone-related artifacts in posterior inferior cerebellar artery (PICA), superior cerebellar artery (SCA), and posterior cerebral artery (PCA) territories. The findings of the present study supporting effectiveness of ASL are consistent with previous studies done in subjects with acute and subacute stroke [6,21,27].

Using a prototype single-slice pulsed ASL sequence, Siewert et al. observed that ASL detected perfusion abnormalities in 18 subacute patients with stroke in comparison with gadolinium-enhanced DSC imaging [28]. Viallon et al. also observed identical results in a group of 41 patients with acute stroke within two weeks of symptom onset [29]. Their results showed that ASL can identify territorial hypo-perfusion in correspondence with DSC; however, for lacunar infarctions, the spatial resolution of ASL was not sufficient to predict local perfusion deficits. These findings are in line with the results of the present study that ASL did not identify perfusion deficits in subjects with smaller infarct size or area as defined with DSC. Hence for small perfusion deficits, ASL is relatively insensitive.

In ASL, cerebral blood flow determines the perfusion weighted images while ischemic tissue is reflected by loss of signal. But in DSC, the lesion detection in stroke is done by the measured mean transit times, derived by the administered bolus. There will be increased transit times in ischemia while a lesion is identified by increased signal or hypointensity.

When comparing both techniques, this difference is important, because the contrast-to-noise of the ASL perfusion-weighted maps is lower and ischemic lesions are less clearly delineated. A relatively long 3.5-minute ASL sequence was used in this study to get a higher signal-to-noise ratio (SNR) image. Perfusion maps can be acquired in less than two minutes by combining pseudo-continuous ASL with background suppression and a single-shot 3-dimensional gradient and spin echo (GRASE) readout. In our study, DSC imaging was not performed in 20.5% of the MR diagnosed cases of ischemic stroke patients due to increased risk of developing nephrogenic systemic fibrosis. Hence, in these patients, like subjects with poor glomerular filtration rate or on hemodialysis, ASL can be a potential viable alternative.

ASL MR perfusion is a rapid noninvasive imaging technique in acute stroke that can demonstrate the ischemic penumbra when combined with diffusion imaging. ASL MR perfusion can depict large perfusion deficits and perfusion-diffusion mismatches in correspondence with DSC (predominantly MCA territory).

ASL technique is not without limitations. It has a lower SNR technique than DSC. ASL is predominantly sensitive to gray matter perfusion and difficult to detect small cerebral blood flow changes in white matter. ASL is more susceptible to motion artefacts. Artefacts can create confusion in acute stroke given that territorial infarcts might be expected to present with identical findings. The present study is also limited by the unavailability of a high magnetic field strength MR scanner (3 Tesla) and quantification software for ASL parameters.

## Conclusions

A rapid ASL MR perfusion scan can be adequate for screening patients with acute stroke with contraindications to gadolinium-based contrast agents. Thus ASL has the potential to replace CT perfusion/MRI DSC perfusion in the future imaging diagnostic work-up for stroke. However, further studies are required to validate its role as the first-line imaging for stroke therapy. ASL when combined with other sequences could make MRI the one-stop imaging solution for stroke assessment in the foreseeable future.
